# Deep Ensemble Learning for the Automatic Detection of Pneumoconiosis in Coal Worker’s Chest X-ray Radiography

**DOI:** 10.3390/jcm11185342

**Published:** 2022-09-12

**Authors:** Liton Devnath, Suhuai Luo, Peter Summons, Dadong Wang, Kamran Shaukat, Ibrahim A. Hameed, Fatma S. Alrayes

**Affiliations:** 1 School of Information and Physical Sciences, The University of Newcastle, Callaghan, NSW 2308, Australia; 2 British Columbia Cancer Research Centre, Vancouver, BC V5Z 1L3, Canada; 3 Quantitative Imaging, CSIRO Data61, Marsfield, NSW 2122, Australia; 4 Department of Data Science, University of the Punjab, Lahore 54890, Pakistan; 5 Department of ICT and Natural Sciences, Norwegian University of Science and Technology, 7491 Trondheim, Norway; 6 Information Systems Department, College of Computer and Information Sciences, Princess Nourah bint Abdulrahman University (PNU), P.O. Box 84428, Riyadh 11671, Saudi Arabia

**Keywords:** ensemble learning, pneumoconiosis, transfer learning, deep learning, chest X-ray radiographs, cross validation, weighted-averaging, majority voting, LOOCV, CheXNet

## Abstract

Globally, coal remains one of the natural resources that provide power to the world. Thousands of people are involved in coal collection, processing, and transportation. Particulate coal dust is produced during these processes, which can crush the lung structure of workers and cause pneumoconiosis. There is no automated system for detecting and monitoring diseases in coal miners, except for specialist radiologists. This paper proposes ensemble learning techniques for detecting pneumoconiosis disease in chest X-ray radiographs (CXRs) using multiple deep learning models. Three ensemble learning techniques (simple averaging, multi-weighted averaging, and majority voting (MVOT)) were proposed to investigate performances using randomised cross-folds and leave-one-out cross-validations datasets. Five statistical measurements were used to compare the outcomes of the three investigations on the proposed integrated approach with state-of-the-art approaches from the literature for the same dataset. In the second investigation, the statistical combination was marginally enhanced in the ensemble of multi-weighted averaging on a robust model, CheXNet. However, in the third investigation, the same model elevated accuracies from 87.80 to 90.2%. The investigated results helped us identify a robust deep learning model and ensemble framework that outperformed others, achieving an accuracy of 91.50% in the automated detection of pneumoconiosis.

## 1. Introduction

Deep learning models are susceptible to noise in training data, as they learn by using stochastic gradient functions. This causes variance errors and may cause overfitting, resulting in low generalisations for validating data. A machine learning technique known as ensemble learning reduces predictive variance by combining the predictions of integrated models. Ensembles are often more accurate than individual classifiers that produce them [1,2,3,4,5].

On the other hand, a deep convolutional neural networks (CNNs) drive process is a difficult optimising process that often does not converge. As a result, CNN’s latest drive weights may not show a consistent or optimal performance as the final model weights. To overcome this problem, the average performance of training weights is calculated as many points in the training cycle [6,7,8]. In general, it could be called the average weight prediction based on the method developed by Polyak-Ruppert [9,10].

Additionally, every CNN is very sensitive to the volume of training data. The model will learn better if you have high-volume data. A special case of cross-validation is called leave-one-out cross-validation (LOOCV), and it is used to evaluate the efficiency of machine learning models with a small dataset. This is a lengthy and costly process, even though it provides a reliable and impartial estimate of model performance. While very simple in application, there are some limitations in using, as there is no need for its application if a large dataset or mathematically costly method is used. During the application of the LOOCV process, each machine learning model is adjusted at a higher number of times, representing a more robust assessment since each data can participate as the entire test dataset [11,12].

In recent years, deep transfer learning with an ensemble of multiple CNNs has been widely used in medical-image processing [13,14,15,16,17]. The trained deep ensemble learning represents a single hypothesis. Empirically, ensembles yield better results when significant diversity among the models, even on a small dataset. Therefore, many ensemble methods seek to promote diversity among the combined models. An ensemble indicates different techniques, including simple averaging, weighted-averaging, majority voting (MVOT), bagging, boosting, CNN blocks, randomizing, and stacking using multi-model predictions on the same dataset [18,19,20,21].

This paper proposed simple averaging, weighted-averaging, and MVOT techniques to detect pneumoconiosis in coal workers’ chest X-ray radiographs (CXRs). The summary of our list of contributions is as follows:

- We have used databases of posterior-anterior (PA) CXRs collected from various hospitals by the Commonwealth Scientific and Industrial Research Organisation (CSIRO), Sydney, Australia. To overcome the problems associated with small datasets, we assessed proposed ensemble techniques, simple averaging, weighted-averaging, and MVOT using randomised cross-fold-validation (RCFV) and leave-one-out cross-validations (LOOCV) of the original dataset independently.
- In all techniques, transfer learning has been implemented using multiple CNN are namely CheXNet [22], DenseNet-121 [23], Inception-V3 [24], Xception [25], and ResNet50 [26]. We proposed ensemble techniques in three investigations: investigation-1 uses simple averaging on RCFV data, investigation-2 uses weighted averaging on RCFV data, and investigation-3 uses MVOT on LOOCV data.
- Finally, we compared the investigation’s outcomes using five formulas of statistical measurements [27], sensitivity, specificity, accuracy, precision, and F1-Score, with state-of-the-art approaches from the literature for the same dataset and highlighted the efficient CNN model in our dataset.

**Figure 1:**
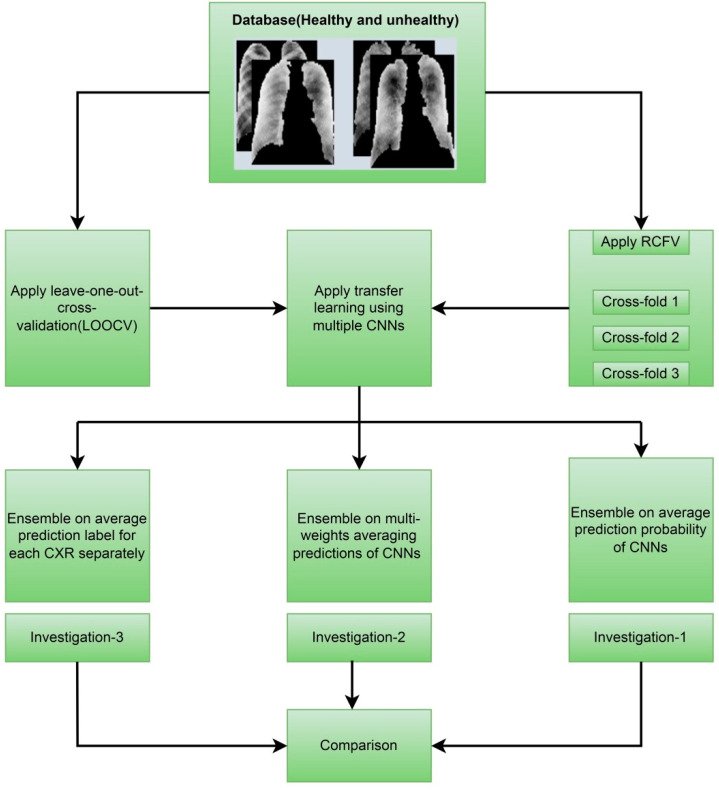
Summary of proposed methodologies in the study of ensemble investigations.

The following Figure 1 depicts the overall contributions, providing an improved understanding of what we have performed in this study. Section 2 presents background studies and findings for pneumoconiosis classification on the same dataset using various classical, traditional machine, and deep learning methods. The orientation of the dataset and the detailed methodologies within each investigation are presented separately in Section 3. Section 4 provides the outcomes of investigation-1, investigation-2, and investigation-3. Section 5 summarised the outcomes of the investigation and compared them with state-of-the-art approaches from the background study for the same dataset. The assumptions and limitations are also highlighted there. Finally, Section 6 provides the conclusion of this research study.

## 2. Background Study

The abnormality on a chest X-ray of the lung is signified by the increase or decrease in density areas. The chest X-ray lung abnormalities with increased density are also known as pulmonary opacities. Pulmonary opacities have three major patterns: consolidation, interstitial, and atelectasis. Among these, the interstitial patterns of pulmonary opacities are mainly responsible for pneumoconiosis disease [28]. According to the International Labour Organization’s (ILO) classification, two abnormalities are observed for all types of pneumoconiosis—parenchymal and pleural. Parenchymal abnormalities are indicated by small opacity shape (round or irregular) and size (1.5 mm < diameter (round) < 10 mm and 1.5 mm < widths (irregular) < 10 mm) and large opacities of a round shape and size less than or equal to 50 mm. Pleural abnormalities are mainly indicated by angle obliteration and the diffusion of thickness in the CXR’s wall [29].

There is no national approach to health screening of coal miners in Australia. In NSW, a chest X-ray is recommended every six years for mine-site workers but it is not mandatory. Medical screening has also failed to detect this potentially fatal disease [30]. For these reasons, it is desirable to develop an established computer-based automatic system further to provide the quantitative evaluation of pneumoconiosis and serve as an initial screening process and a second opinion for medical doctors.

Past research on the automatic classification of pneumoconiosis classical, traditional machine, and deep learning methods were used. The texture features were mostly classified in classical methods using computer- and ILO-based standard classification [31,32,33,34,35,36,37,38,39,40,41]. The profusion of small round opacities and ILO extent properties indicated normal and abnormal lungs. The backpropagation neural networks have been applied to find the shape and size of round opacities from the region of interest (ROI) portions of an image [42,43,44,45]. X-ray abnormalities were categorised and compared with the results of the standard ILO measurement of the size and shape of the round opacities.

In traditional machine learning, different methods for handcrafted feature extraction, or selection were used. The handcrafted features, such as texture features [46,47], from the left –right lung zones [48,49,50,51] were extracted. After the selection of important features, they were input into different machine learning classifiers, such as support vector machine (SVM) [49,52,53,54,55,56,57,58,59,60], decision trees (DT) [55,56], random trees (RT) [57,58,59,60], artificial neural networks (ANNs) [61,62,63], K-nearest neighbours (KNN) [64], self-organizing map (SOM) [64], backpropagation (BP), radial basis function (RBF) neural networks (NN) [57,58,59,60,64,65], and ensemble classifier [49,52,56].

In recent years, deep learning approaches have achieved state-of-the-art results due to their high dimensional feature representation of data [66,67]. Many deep convolutional neural networks performed better than humans, especially in medical image processing [68]. Such examples include identifying indicators for cancer in blood [69] and skin [70,71], malaria in blood cell [72], tuberculosis (TB) from chest X-rays [14,16,73], and more specifically pneumoconiosis in chest X-rays [27,74,75,76,77,78,79,80].

We have conducted different classical, traditional, and deep learning approaches in our previous published works on the same dataset used in this study. We used the ILO Standard Classification System in classical approaches, and the performance is presented in Table 1.

We first extracted handcrafted features using different statistical image analysis methods in traditional machine learning approaches. Then, we input these features into different machine learning classifiers, such as support vector machine (SVM), MLP, NN, K-nearest neighbours (KNN), isolation forest, random forest, and ridge [78]. We show these classifier results in Table 1.

In deep learning approaches, first, we implemented, with and without transfer learning, convolutional neural networks (CNN) to detect pneumoconiosis. Deep transfer learning was implemented using seven pre-trained CNNs, VGG16 [81], VGG19, Inception [24], Xception [25], ResNet50 [26], DenseNet-121 [23], and CheXNet [22]. Then, we performed a performance comparison between them. The comparison was examined using different effects of dropout rates and different augmentation methods used in DL models, with and without transfer learning, to detect pneumoconiosis. We developed a cascade learning model, which outperforms others and achieved an overall classification accuracy of 90.24%, a specificity of 88.46%, and a sensitivity of 93.33% for detecting pneumoconiosis using generated synthesised images from real segmented CXR databases. We have also summarised deep CNNs results in Table 1. The previous studies showed that the deep transfer learning performance of Inception-V3, Xception, ResNet50, DenseNet, and CheXNet was satisfactory compared to classical and traditional approaches.

## 3. Datasets and Methods

The first part of this section discusses our dataset and how it was processed using cross-validation to perform ensemble techniques. In contrast, the rest of the section describes the techniques used in three investigations.

### 3.1. Materials

Out of a collaboration between the University of Newcastle and the Commonwealth Scientific and Industrial Research Organisation (CSIRO) data61 Sydney NSW, Australia, chest X-ray image datasets were built with associated diagnostic labels for this study. CSIRO data61 collected the data from Coal Services Health NSW, St Vincent’s Hospital, Sydney, and Wesley Medical Imaging, Queensland. The publicly available NIOSH teaching chest X-ray dataset and ILO Standard Radiographs (International Labour Organization, (ILO) Genève, Switzerland) were also used to develop parts of the small dataset DL model. All radiographs used in this study are posterior-anterior (PA) radiographs. Seventy-one PA chest radiographs with small parenchymal opacities consistent with pneumoconiosis and 82 PA chest X-rays belonging to normal individuals were used. All data were collected from coal mine workers, including males and females. We conducted ensemble learning using randomised cross-fold-validation and leave-one-out cross-validation. The details are in the following subsections:

#### 3.1.1. Randomised Cross-Fold-Validation

**Figure 2:**
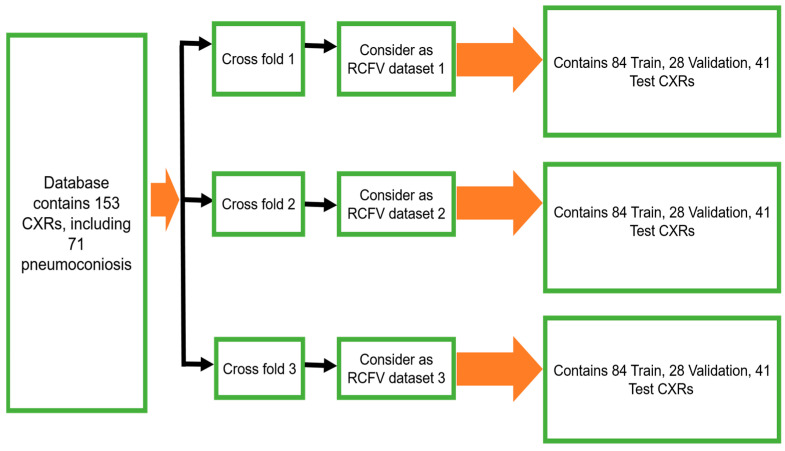
Three RCFVs of our proposed dataset.

To maintain the balance of training data, 112 X-rays (56 normal and 56 pneumoconiosis) were used for training and 41 X-rays (26 normal and 15 pneumoconiosis) were used for testing. Twenty-five percent of training data were kept as a validation dataset for selecting the best model weights based on validation performance. We continued the randomised selection three times and then organised our total dataset into three different folds, namely, as randomised cross-fold-validation (RCFV) dataset 1, dataset 2, and dataset 3, as shown in Figure 2. Therefore, we defined this cross-validation simply as RCFV.

#### 3.1.2. Leave-One-Out Cross-Validation

**Figure 3:**
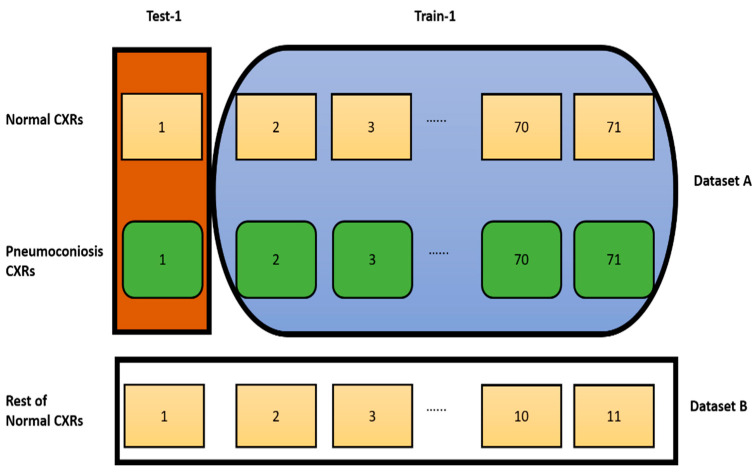
Data orientation for LOOCV implementation.

We proposed a case of cross-validation (LOOCV), which is used to assess the effectiveness of machine learning models with the same dataset. We organised our dataset into two groups, dataset A and dataset B, clearly mentioned in Figure 3. Dataset A contained 71 pairs of images, including an equal number of normal and abnormal (pneumoconiosis) CXRs. Therefore, the remaining 11 normal images were in dataset B. As a result, no correlation exists between the pairs of images.

### 3.2. Methods

The proposed ensemble techniques, simple averaging, weighted averaging, and MVOT, were independently conducted using RCFV and LOOCV datasets. In all techniques, transfer learning was analysed by the same CNNs, namely CheXNet, DenseNet-121, Inception-V3, Xception, and ResNet50. We organized our proposed method into three investigations, as stated below.

#### 3.2.1. Investigation-1: An Ensemble Learning Using Simple Averaging through RCFV Datasets

The deep learning model shows each test element’s probability value within the range [0, 1] during the forecast. Those fractional probabilities are converted to predict class labels using a threshold value condition. An ensemble is mapped using several CNNs model prediction probabilities as a combined decision instead of individually. Therefore, each value of the testing data was predicted by multiple models at once. After that, their average predictive probability interval between [0, 1] indicates the ensemble’s performance.

In this investigation, we implemented deep transfer learning throughout the ensemble using simple averaging of the probability of detection of pneumoconiosis predicted by five CNN models: CheXNet, DenseNet, Inception-V3, Xception, and ResNet50. Afterwards, we calculated the average prediction probabilities on the same RCFV testing datasets 1 to 3, as demonstrated in Figure 2.

CNNs employ a stochastic learning algorithm to optimise training randomly. The optimisation is based on selecting the loss function while the model has been designed. The purpose of the loss function is to determine whether the model is operating properly or incorrectly. The cost function within the CNNs determines the difference in losses between true and predicted values. We applied the regularisation technique to reduce the complexity of a neural network model during training and, thus, prevented overfitting. There are very popular and efficient regularisation techniques called L2. The regularisation term is weighted by the scalar lambda divided by 2 m and added to the regular loss function chosen for the current task. This leads to a new expression for the loss function, as shown in the following Equation (1):

(1) $$Cost\;function=loss\left(binary\_cross\_entropy\right)+\frac{\lambda }{2m}\sum ∥w^{2}∥$$

where *λ* denotes the regularization parameter, and its value may optimise the learning rate for improved predictions. L2 regularisation is also known as the weight decay as it forces the weights to decay towards zero (but not exactly zero).

After taking the output of each of the five models, one GlobalAveragePooling2D layer was added. Three dense layers, with all their output nodes, were connected with all nodes of the next layer. Global Average Pooling is a transaction that computes the average performance of each entity map in the preceding layer. This relatively simple operation helps convert the data into a one-dimensional vector and avoids the overflow of features. There are no trainable parameters, similarly to the Max polling operation.

**Figure 4:**
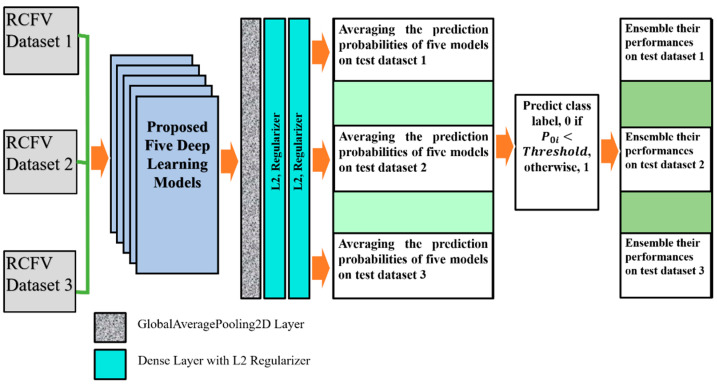
An ensemble learning based on simple averaging of probability prediction values using multiple DL models in three RCFV datasets.

For L2 (0.001), two regularisers were used with the first-two dense layers for better optimisation with the proposed models. The last layer of the classifier used a sigmoid activation function and output probability scores for each class—normal and pneumoconiosis (see Figure 4). We used 512 × 512 X-ray input forms for each proposed CNNs architecture, where the output of the prediction probability value ranged between [0, 1]. The regular loss-function, binary cross-entropy with an Adam optimiser of the learning rate, 0.0001, was also used during training.

We trained each DL model up to 50 epochs and used the last weights to find the prediction probability of normal and abnormal CXRs. For instance, in RCFV dataset-1, we applied five models independently and then calculated their prediction probabilities separately. Next, we calculated the average of their probability values for each unique test image using mathematical Equation (2). If the average value $P_{0i}<\mathrm{Threshold}\left(0.5\right)$, then its predicted label changes to 0; otherwise, it is 1, where i=1 to 26 for normal images and i=1 to 15 for pneumoconiosis images.

(2) $$P_{0i}=\frac{Model1_{0i}+Model2_{0i}+Model3_{0i}+Model4_{0i}+Model5_{0i}}{total\;number\;of\;models}$$

The ensemble performances of five models, CheXNet, DenaseNet, Inception-V3, Xception, and ResNet50, were computed using confusion matrix values, true positives, false negatives, true negatives, and false positives. The ensemble performance for RCFV datasets 2 and 3 was calculated according to the same process used for dataset 1. The details of the proposed workflow are demonstrated in Figure 4. The last three columns illustrate the direction of the average probability forecasts, the forecast labels, and the ensemble performance of the five models across three different cross-datasets.

#### 3.2.2. Investigation-2: An Ensemble Learning Using Weighted Averaging through RCFV Datasets

**Figure 5:**
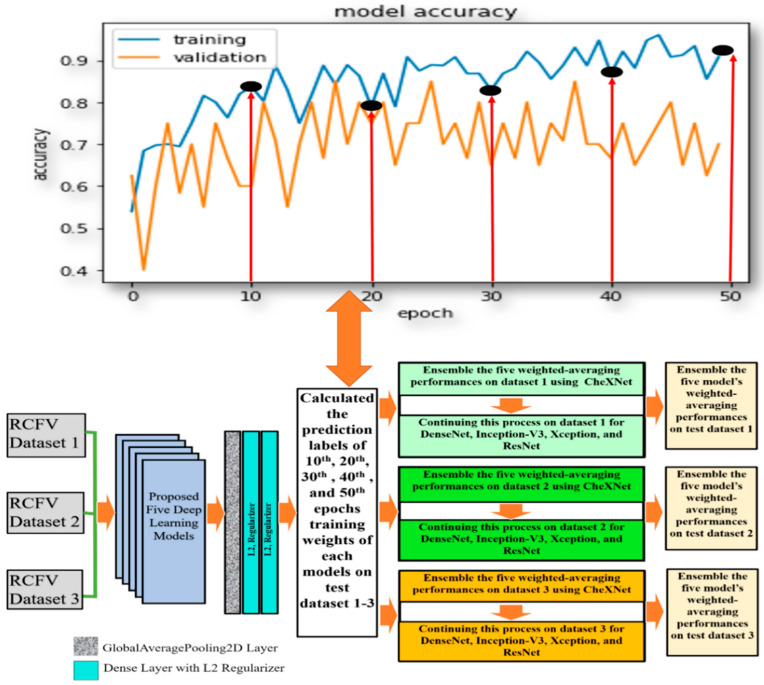
An ensemble using the average prediction probabilities of the combined five DL models on three different RCFV datasets.

With the method used in the previous investigation, we investigated multi-model ensemble learning using the latest drive weights of each model in detecting pneumoconiosis diseases from CXR. We replicate the investigation in this section using the same five models, CheXNet, DenseNet, Inception-V3, Xception, and ResNet50, used in investigation-1. To find the optimal solution for pneumoconiosis detection, we carried out ensemble learning using the combination of the weighted average and majority voting techniques. Here, we focused on its different training epochs in calculating the weighted average ensemble for a single model. We kept the same training process and dataset, as described in investigation-1. In calculating a weighted average on a single model, we used the specified weights from the epochs (10th, 20th, 30th, 40th, and 50th set) for each proposed model, as defined in the central white box in Figure 5.

For instance, in dataset 1, we trained the CheXNet model independently on training data and then computed the five sets of prediction labels of its 10th, 20th, 30th, 40th, and 50th epochs’ weights with test data. The weighted average ensemble prediction labels of the CheXNet were found using the majority voting (MVOT) decision on these five sets of predictions. As a result, if, and only if, the weight of the majority says that a BL image is BL, then the ensemble decision is BL; otherwise, it is normal.

Likewise, we continued this process for dataset 1 for DenseNet-121, Inception-V3, Xception, and ResNet50 models, as described in the second last column in Figure 5. Finally, every single model’s weighted average ensemble return was used to calculate the multi-model ensemble for dataset-1. To accomplish this, MVOT was also applied to the five independent sets of five weighted average prediction labels in the models, as described in the last column of Figure 5.

Similarly, we conducted this process for the testing datasets 2 and 3 and compared weighted-averaging ensemble performances of a single and integrated model using true positive, false negative, true negative, and false positive values from the predicted confusion matrix.

#### 3.2.3. Investigation-3: An Ensemble Learning Using MVOT through the LOOCV Dataset

**Figure 6:**
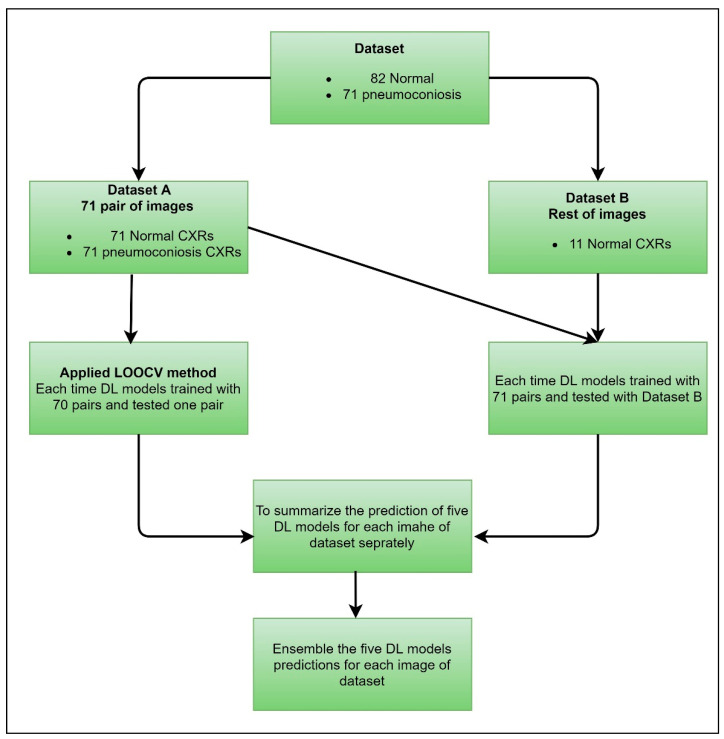
Applying the LOOCV method for the detection of pneumoconiosis using a deep learning algorithm.

In this investigation, we implemented LOOCV to select a robust DL model from CheXNet, DenseNet, InceptionV3, Xception, and ResNet50 by using our organised dataset, as discussed in Section 3.1.2, representing the best competence for detecting detection pneumoconiosis from CXRs. Figure 6 shows how we handled training and testing using DL applications for each cross-data application. For dataset A, every DL model has trained on 70 pairs of images and tested one pair. We continued the process 71 times automatically. We trained the same model with dataset A for dataset B and then tested the performances on dataset B. Next, we independently calculated each DL model performance for each image using a combination of datasets A and B. Finally, each model’s predictions ensemble return is used to calculate the multi-model ensemble for all data in LOOCV using a simple MVOT technique. Therefore, if the majority of models predict as “normal”, then its ensemble prediction is defined as a “normal” or, otherwise, “abnormal” lung.

Finally, we compared the MVOT-based ensemble performances of a single and integrated model using true positive, false negative, true negative, and false positive values from the predicted confusion matrix.

## 4. Results

This section provides a detailed outcome of the three methodological investigations conducted sequentially.

### 4.1. In Investigation-1

We independently applied five deep CNNs models (CheXNet, DenseNet, Inception-V3, Xception, and ResNet50) on RCFV datasets 1–3. The regularisation technique was also implemented for an improved optimisation of CNN learning. We used 84 images (equal class of normal and pneumoconiosis) for training, 28 images (equal class of normal and pneumoconiosis) for validation, and 41 images (26 normal and 15 pneumoconiosis) for testing each model.

We calculated the testing probability of a single image within RCFV datasets 1 to 3. We then converted each fractional value into class label 0 or 1 based on the Threshold (0.5), as shown in Figure 4. Table 2 demonstrates the performance based on the prediction probability of five DL models separately on three different datasets. Each model’s performance was evaluated with the metrics values, including sensitivity, specificity, accuracy, precision, and F1-Score.

In Table 3, Table 4 and Table 5, we demonstrate the five-model prediction probability on the specified columns. Afterwards, we calculated the average prediction value using Equation (2) for each testing image of datasets 1–3. The rightmost two columns represent each image’s predict and true labels separately. The prediction label was calculated based on the average prediction rate of five models using respective test datasets. The true label column indicates that the first 26 and last 15 images were normal and pneumoconiosis classes.

We calculated the confusion metric values, true positive (TP), true negative (TN), false positive (FP), and false negative (FN) for every dataset by counting the predict and true labels from Table 3 to Table 5.

We demonstrated ensemble learning performances on five models’ prediction probabilities using eight evaluation metrics in Table 6, in which the ensemble was learning of the model’s prediction probability represented maximum values of sensitivity, specificity, accuracy, precision, and F1-Score, with a sensitivity of 88.00%, a specificity of 75.00%, an accuracy of 82.93%, a precision of 84.62%, and an F1-score of 86.27% for dataset 2, which are lower values than the individual performances of the CheXNet model without ensemble learning. The performance of ensemble learning using the model’s prediction probability did not improve pneumoconiosis’ detection accuracy.

### 4.2. In Investigation-2

The deep learning models, CheXNet, DenseNet, Inception-V3, Xception, and ResNet50, have been used to calculate the prediction labels of their trained weights of the 10th, 20th, 30th, 40th, and 50th epochs’ iteration, as demonstrated in Figure 5. We used the same training, validation, and testing datasets previously in investigation-1 and evaluated five trained weights and their ensemble performances using the same metrics’ values. All assessments of a single weight and its ensemble were presented separately for each model in the three RCFV cross-fold datasets.

In Table 7, we have represented the CheXNet performances of specified weights with ensemble learning on three RCVF datasets, 1–3. The five trained weights have shown different sensitivity, specificity, accuracy, precision, and F1-Score measurements within three datasets. Ensemble learning shows that the CheXNet achieved a sensitivity of 86.21%, a specificity of 91.67%, an accuracy of 87.80%, a precision of 96.15%, and an F1-score of 90.91% for dataset 1. For dataset 2, CheXNet achieved a sensitivity of 83.87%, a specificity of 100.00%, an accuracy of 87.80%, a precision of 100.00%, and an F1-score of 91.23%. It is also shown that sensitivity of 78.13%, a specificity of 88.89%, an accuracy of 80.49%, a precision of 96.15%, and an F1-score of 86.21% were achieved for dataset 3.

In Table 8, we have represented the DenseNet performances of specified weights with ensemble learning on three RCFV datasets, 1–3. The five trained weights have shown different sensitivity, specificity, accuracy, precision, and F1-Score measurements within the three datasets. Ensemble learning has demonstrated that the DenseNet achieved a sensitivity of 80.77%, a specificity of 66.67%, an accuracy of 75.61%, a precision of 80.77%, and an F1-score of 80.77% for dataset 1. For dataset 2, DenseNet achieved a sensitivity of 79.31%, a specificity of 75.00%, an accuracy of 78.05%, a precision of 88.46%, and an F1-score of 83.64%. It has also shown a sensitivity of 78.57%, a specificity of 69.23%, an accuracy of 75.61%, a precision of 84.62%, and an F1-score of 81.48% achieved for dataset 3.

In Table 9, we demonstrated the InceptionV3 performances of specified weights with ensemble learning on three RCFV datasets, 1–3. The five trained weights have shown different sensitivity, specificity, accuracy, precision, and F1-Score measurements within the three datasets. Ensemble learning has shown that the InceptionV3 achieved a sensitivity of 85.71%, a specificity of 84.62%, an accuracy of 85.37%, a precision of 92.31%, and an F1-score of 88.89% for dataset 1. For dataset 2, the InceptionV3 achieved a sensitivity of 88.89%, a specificity of 85.71%, an accuracy of 87.80%, a precision of 92.31%, and an F1-score of 90.57%. It has also shown that sensitivity of 74.29%, a specificity of 100.00%, an accuracy of 78.05%, a precision of 100.00%, and an F1-score of 85.25% were achieved for dataset 3.

In Table 10, we demonstrated the Xception performances of specified weights with ensemble learning on three RCFV datasets, 1–3. The five trained weights have shown different sensitivity, specificity, accuracy, precision, and F1-Score measurements within the three datasets. Ensemble learning has shown that the Xception achieved a sensitivity of 90.91%, a specificity of 68.42%, an accuracy of 80.49%, a precision of 76.92%, and an F1-score of 83.33% for dataset 1. For dataset 2, Xception achieved a sensitivity of 87.50%, a specificity of 70.59%, an accuracy of 80.49%, a precision of 80.77%, and an F1-score of 84.00%. It has also shown that a sensitivity of 85.00%, a specificity of 57.14%, an accuracy of 70.73%, a precision of 65.38%, and an F1-score of 73.91% were achieved for dataset 3.

In Table 11, we demonstrated the ResNet50 performances of specified weights with ensemble learning on three RCFV datasets, 1–3. The five trained weights have shown different sensitivity, specificity, accuracy, precision, and F1-Score measurements within the three datasets. Ensemble learning has shown that the ResNet50 achieved a sensitivity of 73.08%, a specificity of 53.33%, an accuracy of 65.85%, a precision of 73.08%, and an F1-score of 73.08% for dataset 1. For dataset 2, the ResNet50 achieved a sensitivity of 100.00%, a specificity of 75.00%, an accuracy of 87.80%, a precision of 80.77%, and an F1-score of 89.36%. It has also shown that a sensitivity of 81.48%, a specificity of 71.43%, an accuracy of 78.05%, a precision of 84.62%, and an F1-score of 83.02% were achieved for dataset 3.

In Table 12, we demonstrated the multi-model weighted-averaging ensemble results using the five models’ independent average-weighted ensemble performances from Table 7 to Table 11. Therefore, we compared single-model ensemble learning with multi-model ensemble learning. The performances in Table 12 show that multi-model ensemble learning achieved the same detection accuracy of 82.93% for all datasets. Therefore, this approach did not outperform the model when applied individually. Comparing ensemble learning in individual and combined results shows that the CheXNet model outperformed others and investigation-1.

### 4.3. In Investigation-3

We calculated the true positive, true negative, false positive, and false negative values using the prediction label of each image from dataset A and dataset B, as demonstrated in Figure 6. Then, the performance of the five DL models was evaluated individually with sensitivity, specificity, accuracy, precision, and F1-score, indicating the percentage to which the model correctly identified both normal and pneumoconiosis CXRs. The individual and ensemble performances of the proposed models, CheXNet, DenseNet, InceptionV3, Xception, and ResNet50, are shown in Table 13. The LOOCV method was applied to find the most efficient model with the entire dataset.

Table 13 demonstrates that the proposed ensemble learning achieved the best performances on our dataset. As the most efficient method, CheXNet achieved the maximum accuracy of 90.20%, a sensitivity of 88.51%, specificity of 92.42%, a precision of 93.90%, and an F1-score of 91.12%. The ResNet50 model had the worst performance, and the other models’ performances were not bad. Finally, the proposed ensemble achieved an accuracy of 91.50%, a sensitivity of 90.14%, a specificity of 92.68%, a precision of 91.43%, and an F1-score of 90.78% in our dataset.

## 5. Discussion

From investigation-1 to investigation-3, we applied different methodologies to improve pneumoconiosis detections in CXRs. In Table 14, we summarised the best statistical combination derived from the investigated ensemble techniques. Here, the lower the standard deviation (SD), the closer the values are to the mean of the set of investigations. The higher the SD, the wider the range of investigations. All techniques were processed to find the optimal solution for detecting pneumoconiosis from X-ray images. Investigation-1 had the best combination of accuracy of 82.93%, a sensitivity of 88.00%, a specificity of 75.00%, a precision of 84.62%, and an F1-score 86.27%, as summarised in Table 14, even though these are lower values than the individual performances of the CheXNet model without ensemble learning, as shown in Table 2. When compared to an individual, the performance of the ensemble learning technique in the first investigation did not improve pneumoconiosis detection’s accuracy.

In investigation-2, we found that the detection performances slightly improved in the ensemble of multi-weighted averaging on a single model, CheXNet, as demonstrated in Table 14, which has shown improved statistical combinations than the other methodological findings in investigation-1. In investigation-3, we first observed that the same CheXNet model independently improved the accuracy from 87.80 to 90.20%. In addition, the proposed ensemble learning obtained 91.50% peak performance for detecting pneumoconiosis in coal workers from CXRs with state-of-the-art methods. Investigation-3 had an excellent success rate of more than 90.00% for all five observations. Therefore, as ground truth, our proposed ensemble learning outperformed other state-of-the-art classical and traditional machine and deep learning methods, as summarised in Table 1.

**Figure 7:**
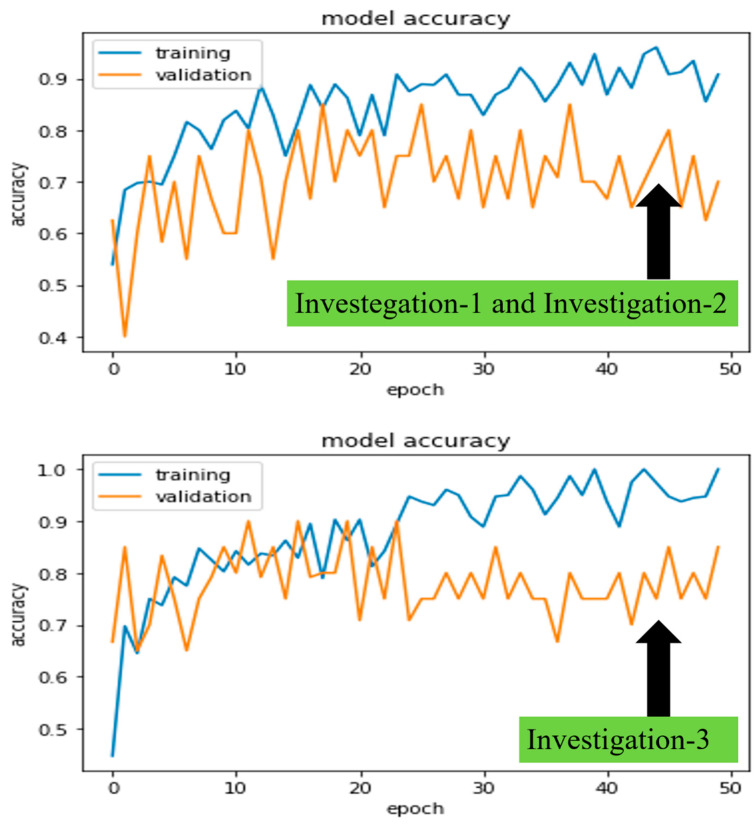
The CheXNet model’s training and validation accuracies in investigation-1 and 2 (on **top**) and investigation-3 (on **bottom**) per epoch.

**Figure 8:**
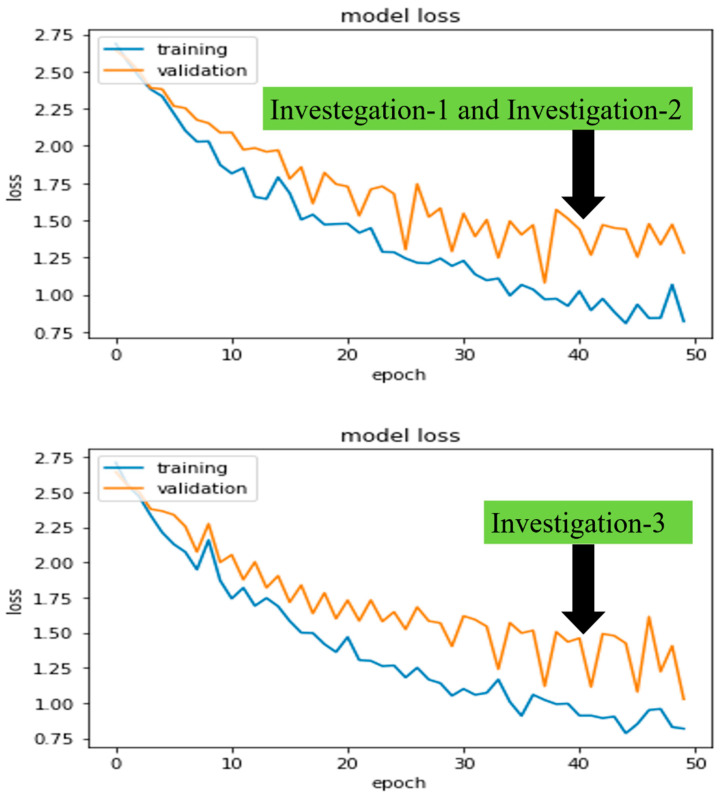
The CheXNet model’s training and validation losses in investigation-1 and 2 (**top**) and investigation-3 (**bottom**) per epoch.

The University of Newcastle’s (Australia) high-performance computing (HPC) system was used for all investigations. Python 3.6 was used to run deep learning platform Keras 2.2.2 and machine learning platform Scikit-learn 0.19.1. In addition, we also looked at how long it took to train five different models, CheXNet, DenseNet, Inception-V3, Xception, and ResNet50, which took 19, 20, 16, 13, and 11 min, respectively, for 50 epochs. Furthermore, the model training and validation performance were monitored from the continuity of average (Avg) and standard deviation (SD) of accuracies and losses on each epoch. Figure 7 and Figure 8 demonstrated the robust model, CheXNet training, and validation accuracies and losses for all proposed investigations. The investigated DL model was validated using Equations (3) and (4), where i indicates the $i^{th}$ epoch’s (N=1 to 50) accuracy or loss values of a trained model. By comparing Avg and SD, we were able to pick the best-trained model to perform the test. In the following paragraphs, we present these values for the same robust model, CheXNet.

(3) $$Avg=\frac{\sum_{i=1}^{N}i}{N}$$

(4) $$SD=\sqrt{\frac{1}{N-1}\overset{N}{\underset{i=1}{\sum }}{\left(i-Avg\right)}^{2}}$$

In investigations 1 and 2, the Avg and SD of training and validation accuracies were calculated as ${\mathrm{Avg}}_{\mathrm{Training}}=0.86$, $Avg_{validation}=0.75$, ${\mathrm{SD}}_{\mathrm{training}}=0.09$, and ${\mathrm{SD}}_{\mathrm{validation}}=0.05$ approximately. Similarly, losses were calculated as ${\mathrm{Avg}}_{\mathrm{Training}}=0.78$, $Avg_{validation}=0.97$, ${\mathrm{SD}}_{\mathrm{training}}=0.39$, and ${\mathrm{SD}}_{\mathrm{validation}}=0.24$ approximately.

Finally, in investigation 3, the Avg and SD of training and validation accuracies were calculated as ${\mathrm{Avg}}_{\mathrm{Training}}=0.88$, $Avg_{validation}=0.78$, ${\mathrm{SD}}_{\mathrm{training}}=0.12$, and ${\mathrm{SD}}_{\mathrm{validation}}=0.08$ approximately. Similarly, losses were calculated as ${\mathrm{Avg}}_{\mathrm{Training}}=0.7$, $Avg_{validation}=0.91$, ${\mathrm{SD}}_{\mathrm{training}}=0.33$, and ${\mathrm{SD}}_{\mathrm{validation}}=0.18$ approximately.

The de-identified private CXRs database was gathered from the Coal Services Health NSW, St Vincent’s Hospital, Sydney, Wesley Medical Imaging, Queensland, and ILO standard, which are supposed to comprise 100% correct assumptions for this research study. However, our proposed ensemble technique achieved an accuracy of 91.50%, a true positive rate (sensitivity) of 90.14%, and a true negative rate (specificity) of 92.68%, which were, on average, 10% lower than our assumptions.

This research study has a few limitations as well. First and foremost, the CSIRO’s Sydney, Australia, office anonymised this private dataset, which cannot be accessed without their written consent [77]. However, if the proposed dataset is large, the outperformed ensemble investigation-3 may be mathematically expensive and take longer to obtain a robust assessment than other investigations. Future studies will focus on testing the proposed model in a clinical setting and gathering input to improve the methodology further. Furthermore, we also recommend some form of variations in tool coupling to at least retain the best case.

## 6. Conclusions

In the paper, deep ensemble learning techniques were applied to detect pneumoconiosis automatically in the CXRs of coal workers. The ensemble was exploited by analysing the average probability, multi-weighted averaging, and majority label predictions using five deep learning models by using randomised cross-folds and leave-one-out cross-validations datasets. Three investigated results indicate the most efficient model, CheXNet on our small dataset that improves the accuracy from 85.37 to 90.20% independently. The integrated ensemble techniques with deep learning methods outperformed others, achieving an accuracy of 91.50% in the automated detection of pneumoconiosis. This study can be beneficial to researchers in the computer-aided diagnostic (CAD) system and to researchers dealing with small datasets in a real-time environment. Moreover, these investigations are useful for locating a reliable approach among the numerous alternatives. The approach substantially impacts clinical studies and is significant to physicians and other healthcare professionals.

## Figures and Tables

**Table 1:** Summary of all classical, traditional, and deep learning approaches previously performed on the same dataset.

Year	Ref No.	Dataset	Classification Approaches	Evaluation Performance		
				Accuracy	Specificity	Recall
2019	[78]	Same dataset that was used in this paper	Classical method, ILO standard	83.00%	81.70%	84.60%
2019	[78]		Traditional machine learning classifiers	SVM = 73.17%	92.31%	73.30%
				MLP = 71.11%	72.00%	70.00%
				NN = 83.00%	85.00%	82.00%
				Isolation Forest = 73.30%	92.31%	73.17%
				KNN = 69.30%	-	-
				Random Forest= 70.80%	-	-
				Ridge = 76.90%	87.00%	63.00%
2019	[76]		CNN-without transfer learning	DenseNet = 80.49%	66.67%	88.46%
2020	[77]	153 CXR including 71 pneumoconiosis	Deep CNN-transfer learning	VGG16 = 82.93%	80.00%	84.62%
				VGG19 = 80.49%	80.00%	80.77%
				ResNet = 85.37%	80.00%	88.46%
				InceptionV3 = 87.80%	86.67%	88.46%
				Xception = 85.37%	93.33%	80.77%
				DenseNet = 82.93%	80.00%	84.62%
				CheXNet = 85.37%	93.33%	80.77%
2019	[75]		Cascaded Learning	90.24%	88.46%	93.33%

**Table 2:** The performance is based on the prediction probability of five CNNs models separately on three RCFV datasets.

RCFV Dataset	Model	Sensitivity (%)	Specificity (%)	Accuracy (%)	Precision (%)	F1-Score (%)
1	**CheXNet**	**83.33**	**90.91**	**85.37**	**96.15**	**89.29**
	DenseNet	84.00	68.75	78.05	80.77	82.35
	InceptionV3	76.47	100.00	80.49	100.00	86.67
	Xception	71.88	66.67	70.73	88.46	79.31
	Resnet50	71.43	53.85	65.85	76.92	74.50
2	**CheXNet**	**83.33**	**90.91**	**85.37**	**96.15**	**89.29**
	DenseNet	78.57	69.23	75.61	84.62	81.48
	InceptionV3	82.14	76.92	80.49	88.46	85.19
	Xception	90.91	68.42	80.49	76.92	83.33
	Resnet50	100.00	60.00	75.61	61.54	76.19
3	**CheXNet**	**80.00**	**81.82**	**80.49**	**92.31**	**85.71**
	DenseNet	72.00	50.00	63.41	69.23	70.59
	InceptionV3	73.53	85.71	75.61	96.15	83.33
	Xception	85.71	60.00	73.17	69.23	76.60
	Resnet50	75.00	77.78	75.61	92.31	82.76

**Table 3:** Average testing probabilities of five models on RCFV dataset 1.

Testing Img No.	CheXNet	DenseNet	Inception V3	Xception	ResNet50	Average of Five Models
1	0.932637	0.040125	0.450016	0.025675	0.002168	0.290125
2	0.105605	0.007986	0.028912	0.002934	0.000006	0.029089
3	0.039132	0.257459	0.089236	0.032493	0.000001	0.083665
4	0.052019	0.325931	0.162149	0.227888	0.000924	0.153783
5	0.036418	0.462622	0.294643	0.727933	0.999364	0.504196
6	0.124626	0.002598	0.092452	0.001123	0.000331	0.044227
7	0.122279	0.004001	0.055866	0.000066	0.000010	0.036445
8	0.238593	0.001707	0.064059	0.014911	0.000269	0.063908
9	0.178124	0.976175	0.122117	0.839697	0.999989	0.623221
10–41	0.336297	0.459394	0.277296	0.257559	0.379978	0.342105

**Table 4:** Average testing probabilities of five models on RCFV dataset 2.

Testing Img No.	CheXNet	DenseNet	InceptionV3	Xception	ResNet50	Average of Five Models
1	0.272361	0.001067	0.000956	0.028802	0.001717	0.060981
2	0.579127	0.877206	0.837060	0.994562	0.999967	0.857585
3	0.360972	0.002431	0.001269	0.007587	0.632691	0.200990
4	0.377410	0.000500	0.014508	0.529541	0.847036	0.353799
5	0.418817	0.008354	0.004357	0.000794	0.303923	0.14725
6	0.470152	0.000417	0.002373	0.001013	0.209452	0.136682
7	0.113039	0.005697	0.016339	0.984138	0.387843	0.301412
8	0.364287	0.007771	0.000192	0.010377	0.007184	0.077963
9	0.223685	0.003317	0.596456	0.956602	0.977765	0.551566
10–41	0.417755	0.395281	0.362951	0.464778	0.661663	0.460483

**Table 5:** Average testing probabilities of five models on RCFV dataset 3.

Testing Img No.	CheXNet	DenseNet	InceptionV3	Xception	ResNet50	Average of Five Models
1	0.669168	0.099058	0.191127	0.981978	0.161130	0.420493
2	0.387482	0.002331	0.011922	0.001326	0.000510	0.080715
3	0.334752	0.218163	0.033087	0.002177	0.000000	0.117636
4	0.425194	0.985000	0.265910	0.996532	0.716426	0.677813
5	0.192840	0.998790	0.241946	0.997667	0.996475	0.685544
6	0.455117	0.805958	0.013506	0.000174	0.000158	0.254983
7	0.431945	0.279125	0.009881	0.000019	0.000255	0.144245
8	0.530023	0.673950	0.136910	0.250284	0.005652	0.319364
9	0.464333	0.000178	0.001372	0.000228	0.000017	0.093226
10–41	0.431321	0.376997	0.244273	0.524577	0.228047	0.361043

**Table 6:** An ensemble using the average prediction probabilities of combining five DL models on three RCFV datasets.

RCFV Dataset	Ensemble of Models	Sensitivity (%)	Specificity (%)	Accuracy (%)	Precision (%)	F1-Score (%)
1	CheXNet, DenseNet, InceptionV3, Xception, Resnet50	69.70	62.50	68.29	88.46	77.97
2		88.00	75.00	82.93	84.62	86.27
3		82.14	76.92	80.49	88.46	85.19

**Table 7:** The CheXNet performances of specified weights with ensemble learning.

RCFV Dataset	CheXNet Trained Weights	Sensitivity (%)	Specificity (%)	Accuracy (%)	Precision (%)	F1-Score (%)
1	10-epoch	88.89	85.71	87.8	92.31	90.57
	20-epoch	80.65	90.00	82.93	96.15	87.72
	30-epoch	83.33	90.91	85.37	96.15	89.29
	40-epoch	83.33	90.91	85.37	96.15	89.29
	50-epoch	83.33	90.91	85.37	96.15	89.29
	**Ensemble learning**	**86.21**	**91.67**	**87.80**	**96.15**	**90.91**
2	10-epoch	80.77	66.67	75.61	80.77	80.77
	20-epoch	83.33	90.91	85.37	96.15	89.29
	30-epoch	80.00	81.82	80.49	92.31	85.71
	40-epoch	74.29	100.00	78.05	100.00	85.25
	50-epoch	72.22	100.00	75.61	100.00	83.87
	**Ensemble learning**	**83.87**	**100.00**	**87.80**	**100.00**	**91.23**
3	10-epoch	80.00	81.82	80.49	92.31	85.71
	20-epoch	73.53	85.71	75.61	96.15	83.33
	30-epoch	71.43	83.33	73.17	96.15	81.97
	40-epoch	71.43	83.33	73.17	96.15	81.97
	50-epoch	67.57	75.00	68.29	96.15	7937
	**Ensemble learning**	**78.13**	**88.89**	**80.49**	**96.15**	**86.21**

**Table 8:** DenseNet performances of specified weights with ensemble learning.

RCFV Dataset	DenseNet Trained Weights	Sensitivity (%)	Specificity (%)	Accuracy (%)	Precision (%)	F1-Score (%)
1	10-epoch	80.77	66.67	75.61	80.77	80.77
	20-epoch	72.22	100.00	75.61	100.00	83.87
	30-epoch	76.67	72.73	75.61	88.46	82.14
	40-epoch	85.00	57.14	70.73	65.38	73.91
	50-epoch	84.00	68.75	78.05	80.77	82.35
	**Ensemble learning**	**80.77**	**66.67**	**75.61**	**80.77**	**80.77**
2	10-epoch	86.96	66.67	78.05	76.92	81.63
	20-epoch	95.45	73.68	85.37	80.77	87.50
	30-epoch	76.47	100.00	80.49	100.00	86.67
	40-epoch	80.65	90.00	82.93	96.15	87.72
	50-epoch	78.57	69.23	75.61	84.62	81.48
	**Ensemble learning**	**79.31**	**75.00**	**78.05**	**88.46**	**83.64**
3	10-epoch	75.00	77.78	75.61	92.31	82.76
	20-epoch	72.22	100.00	75.61	100.00	83.87
	30-epoch	80.00	62.50	73.17	76.92	78.43
	40-epoch	72.00	50.00	63.41	69.23	70.59
	50-epoch	80.00	62.50	73.17	76.92	78.43
	**Ensemble learning**	**78.57**	**69.23**	**75.61**	**84.62**	**81.48**

**Table 9:** The InceptionV3 performances of specified weights with ensemble learning.

RCFV Dataset	InceptionV3 Trained Weights	Sensitivity (%)	Specificity (%)	Accuracy (%)	Precision (%)	F1-Score (%)
1	10-epoch	88.00	75.00	82.93	84.62	86.27
	20-epoch	82.14	76.92	80.49	88.46	85.19
	30-epoch	82.14	76.92	80.49	88.46	85.19
	40-epoch	94.12	58.33	73.17	61.54	74.42
	50-epoch	76.47	100.00	80.49	100.00	86.67
	**Ensemble learning**	**85.71**	**84.62**	**85.37**	**92.31**	**88.89**
2	10-epoch	85.71	84.62	85.37	92.31	88.89
	20-epoch	85.71	84.62	85.37	92.31	88.89
	30-epoch	88.46	80.00	85.37	88.46	88.46
	40-epoch	76.47	100.00	80.49	100.00	86.67
	50-epoch	82.14	76.92	80.49	88.46	85.19
	**Ensemble learning**	**88.89**	**85.71**	**87.80**	**92.31**	**90.57**
3	10-epoch	74.19	70.00	73.17	88.46	80.70
	20-epoch	72.73	75.00	73.17	92.31	81.36
	30-epoch	6842	100.00	70.73	100.00	81.25
	40-epoch	73.53	85.71	75.61	96.15	83.33
	50-epoch	73.53	85.71	75.61	96.15	83.33
	**Ensemble learning**	**74.29**	**100.00**	**78.05**	**100.00**	**85.25**

**Table 10:** The Xception performance of specified weights with ensemble learning.

RCFV Dataset	Xception Trained Weights	Sensitivity (%)	Specificity (%)	Accuracy (%)	Precision (%)	F1-Score (%)
1	10-epoch	94.12	58.33	73.17	61.54	74.42
	20-epoch	94.44	60.87	75.61	65.38	77.27
	30-epoch	84.21	54.55	68.29	61.54	71.11
	40-epoch	77.78	64.29	73.17	80.77	79.25
	50-epoch	71.88	66.67	70.73	88.46	79.31
	**Ensemble learning**	**90.91**	**68.42**	**80.49**	**76.92**	**83.33**
2	10-epoch	91.67	76.47	85.37	84.62	88.00
	20-epoch	90.48	65.00	78.05	73.08	80.85
	30-epoch	88.00	75.00	82.93	84.62	86.27
	40-epoch	85.19	78.57	82.93	88.46	86.79
	50-epoch	82.14	76.92	80.49	88.46	85.19
	**Ensemble learning**	**87.50**	**70.59**	**80.49**	**80.77**	**84.00**
3	10-epoch	81.82	57.89	70.73	69.23	75.00
	20-epoch	88.89	56.52	70.73	61.54	72.73
	30-epoch	85.71	60.00	73.17	69.23	76.60
	40-epoch	81.82	57.89	70.73	69.23	75.00
	50-epoch	76.00	56.25	68.29	73.08	74.51
	**Ensemble learning**	**85.00**	**57.14**	**70.73**	**65.38**	**73.91**

**Table 11:** The ResNet50 performances of specified weights with ensemble learning.

RCFV Dataset	ResNet50 Trained Weights	Sensitivity (%)	Specificity (%)	Accuracy (%)	Precision (%)	F1-Score (%)
1	10-epoch	65.79	66.67	65.85	96.15	78.13
	20-epoch	75.00	61.54	70.73	80.77	77.78
	30-epoch	73.08	53.33	65.85	73.08	73.08
	40-epoch	70.59	41.67	53.66	46.15	55.81
	50-epoch	71.43	53.85	65.85	76.92	74.07
	**Ensemble learning**	**73.08**	**53.33**	**65.85**	**73.08**	**73.08**
2	10-epoch	100.00	55.56	70.73	53.85	70.00
	20-epoch	86.96	66.67	78.05	76.92	81.63
	30-epoch	88.89	85.71	87.80	92.31	90.57
	40-epoch	100.00	60.00	75.61	61.54	76.19
	50-epoch	95.65	77.78	87.80	84.62	89.80
	**Ensemble learning**	**100.00**	**75.00**	**87.80**	**80.77**	**89.36**
3	10-epoch	75.00	61.54	70.73	80.77	77.78
	20-epoch	73.33	70.73	63.64	84.62	78.57
	30-epoch	86.36	63.16	75.61	73.08	79.17
	40-epoch	75.00	77.78	75.61	92.31	82.76
	50-epoch	73.53	85.71	75.61	96.15	83.33
	**Ensemble learning**	**81.48**	**71.43**	**78.05**	**84.62**	**83.02**

**Table 12:** Final ensemble learning uses multi-weighted DL models on three different datasets.

RCFV Dataset	Ensemble of Models	Sensitivity (%)	Specificity (%)	Accuracy (%)	Precision (%)	F1-Score (%)
1	CheXNet, DenseNet, InceptionV3, Xception, Resnet50	88.00	75.00	82.93	84.62	86.27
2		88.00	75.00	82.93	84.62	86.27
3		80.65	90.00	82.93	96.15	87.72

**Table 13:** The performance of the leave-one-out method with five DL models.

Dataset	Efficiency Measurement	Model	Sensitivity (%)	Specificity (%)	Accuracy (%)	Precision (%)	F1-Score (%)
Contains 153 CXRs, including 71 Pneumoconiosis	Individually	CheXNet	**88.51**	**92.42**	**90.20**	**93.90**	**91.12**
		DenseNet	88.89	86.11	87.58	87.80	88.34
		InceptionV3	87.06	88.24	87.58	90.24	88.62
		Xception	85.88	86.76	86.27	89.02	87.43
		Resnet50	82.76	84.85	83.66	87.80	85.21
	Ensemble of five model’s predictions		**90.14**	**92.68**	**91.50**	**91.43**	**90.78**

**Table 14:** Summary of best statistical combination achieved using the proposed techniques.

Techniques	Sensitivity (%)	Specificity (%)	Accuracy (%)	Precision (%)	F1-Score (%)
Investigation-1	88.00	75.00	82.93	84.62	86.27
Investigation-2	86.21	91.67	87.80	96.15	90.91
Investigation-3	**90.14**	**92.68**	**91.50**	**91.43**	**90.78**
Mean	88.12	86.45	87.41	90.73	89.32
SD	1.61	8.11	3.51	4.73	2.16
